# Long-term association of vegetable and fruit intake with risk of dementia in Japanese older adults: the Hisayama study

**DOI:** 10.1186/s12877-022-02939-2

**Published:** 2022-03-28

**Authors:** Yasumi Kimura, Daigo Yoshida, Tomoyuki Ohara, Jun Hata, Takanori Honda, Yoichiro Hirakawa, Mao Shibata, Emi Oishi, Satoko Sakata, Yoshihiko Furuta, Sanmei Chen, Kazuhiro Uchida, Tomohiro Nakao, Takanari Kitazono, Toshiharu Ninomiya

**Affiliations:** 1 Center for Cohort Studies, Graduate School of Medical Sciences, Kyushu University, Fukuoka, Japan; 2 Department of Epidemiology and Public Health, Graduate School of Medical Sciences, Kyushu University, 3-1-1 Maidashi, Higashi-ku, Fukuoka, 812-8582 Japan; 3 Division of Community Health and Home Care Nursing, Department of Nursing, Faculty of Nursing, Fukuoka Nursing College, Fukuoka, Japan; 4 Department of Neuropsychiatry, Graduate School of Medical Sciences, Kyushu University, Fukuoka, Japan; 5 Department of Medicine and Clinical Science, Graduate School of Medical Sciences, Kyushu University, Fukuoka, Japan; 6 Department of Global Health Nursing, Graduate School of Biomedical and Health Sciences, Hiroshima University, Hiroshima, Japan; 7 Department of Nutritional Sciences, Nakamura Gakuen University, Fukuoka, Japan

**Keywords:** Vegetable intake, Fruit intake, Dementia, Alzheimer’s disease, Vascular dementia, Elderly, Cohort studies, Japanese

## Abstract

**Background:**

Several prospective Western studies have reported an inverse association of vegetable and fruit intake with dementia risk. However, there is limited epidemiologic evidence in Asians. This study investigated the association of intakes of vegetables, fruits, and their nutrients on the risk of incident dementia and its subtypes in a Japanese community.

**Methods:**

A total of 1071 participants (452 men and 619 women) aged ≥60 years without dementia at baseline were prospectively followed up for 24 years. Intakes of vegetables, fruits, and nutrients were evaluated using a 70-item semiquantitative food frequency questionnaire at baseline and were categorized into quartiles separately by gender. The outcome measure was the development of dementia and its subtypes—namely, Alzheimer’s disease (AD) and vascular dementia (VaD). The risk estimates of incident dementia were computed using a Cox proportional hazards model.

**Results:**

During the long-term follow-up period, 464 subjects developed dementia, of whom 286 had AD and 144 had VaD. Higher vegetable intake was associated gradually with lower risk of developing dementia and AD (both *P*-trend < 0.05), but not VaD, after adjusting for confounders. Subjects allocated the highest quartile of vegetable intake had 27 and 31% lower risk of dementia and AD, respectively, than those with the lowest quartile. The risk of dementia decreased significantly with higher intakes of vitamin A, riboflavin, vitamin C, magnesium, calcium, and potassium (all *P*-trend < 0.05). Subjects with higher total dietary fiber intake tended to be at decreased risk for total dementia (*P*-trend = 0.07). Meanwhile, there were no significant associations between fruit intake and the risk of dementia and its subtypes.

**Conclusion:**

Higher intakes of vegetables and their constituent nutrients were associated with a lower risk of dementia in Japanese older adults. A diet rich in vegetables may be beneficial in reducing the dementia risk in Asians.

**Supplementary Information:**

The online version contains supplementary material available at 10.1186/s12877-022-02939-2.

## Background

Dementia is a common public health problem [1]. The number of subjects with dementia has been increasing worldwide: approximately 47 million people had dementia in 2015, and this number is expected to increase to 132 million by 2050 [1]. Alzheimer’s disease (AD) is the most common type of dementia in humans, representing approximately 60–70% of all cases [2]. In Japan, it has also been reported that the prevalence and incidence of AD has increased with time due to the aging population [3, 4], but these upward trends were observed even after adjusting for age and sex, suggesting the involvement of some other factor(s) in addition to aging [4]. To reduce the burden of dementia, it is essential to clarify the etiology and risk factors for AD and establish effective strategies for preventing dementia. In this context, diet is considered to be an important modifiable lifestyle factor for reducing the risk of dementia [5, 6]. Our study group previously reported a dietary pattern associated with reduced risk of dementia, which was characterized by a high intake of vegetables, fruits, soybeans and soybean products, algae, and milk and dairy products and a low intake of rice, in the Japanese older adults [7]. However, the amount of intake of each food group that was associated with reduced risk of dementia was not addressed in the previous study. Moreover, vegetables and fruits are relatively rich in vitamins, minerals and other bioactive compounds in addition to being good sources of dietary fiber [8]. Intakes of these nutrients have been considered to be associated with lower risk of cognitive impairment [9–14]. Hence, the present study focused on the quantities of vegetables and fruits and their nutrients consumed as part of a typical healthy diet [15] that are associated with a lower risk of dementia.

Several prospective studies conducted in Western countries have reported that subjects consuming high proportions of vegetables and fruits had a low risk of dementia [16–18]. The incidence of dementia in African countries (total population > 60 years, crude estimated prevalence of dementia: 2.6%) is lower than that in Western and Asian countries (6.2% in Europe and 6.5% in America, 3.9% in Asia) [19], but no association between fruits and vegetables and dementia has been found [20]. This is due to limitations such as small studies and poor methodology [19, 20]. For Asian populations, on the other hand, there has been only one prospective study investigating this issue [21]. Since Asian populations have different race and lifestyle backgrounds from Western populations, this issue should be further addressed in Asian populations. Certainly, a majority of the world’s countries, and especially Western and African nations, have reported that vegetable intakes in their populations are lower than the 240 g/day currently recommended by the World Health Organization [22], whereas vegetable intake is relatively high in East Asian countries [23]. Vegetable intake in Japan is 281 g/day, also higher than the World Health Organization recommendation [24]. Therefore, we considered that it would be worthwhile to examine the association of vegetable intake on the risk of dementia in a population with high vegetable intake. Moreover, most of the above-mentioned epidemiological studies have combined vegetable and fruit intake and examined the association between these intakes and the risk of dementia [16–18]. Similar nutrients are included in vegetables and fruits, but the amount of each nutrient is different betwen vegetable and fruit. Thus, it would be preferable to examine the association of vegetable intake separately from that of fruit intake, since vegetables and fruits contain different nutrients.

The aim of this study was to separately examine the association of vegetable intake and fruit intake with the development of dementia and its subtypes in a prospective cohort study of older Japanese adults. In addition, we also assessed the association of individual nutrients of vegetables and fruits with the risk of dementia.

## Methods

### Study population

The Hisayama study is an ongoing prospective cohort study performed in the town of Hisayama, which is located in a suburb of the Fukuoka metropolitan area, Japan [25]. This study was begun in 1961 to examine the prevalence and incidence of cerebrocardiovascular diseases and dementia and their risk factors in Japanese. Full community surveys of the health status and neurological conditions of residents aged 40 and older have been repeated every 1 to 2 years since 1961 [25, 26]. Comprehensive surveys of cognitive impairment and dementia in the elderly, including neuropsychological tests, have been performed since 1985 [4, 25]. In 1988, a follow-up population was formed as a baseline, and 1228 residents aged 60 and older (participation rate: 90.3%) underwent a screening examination for the current study. After exclusion of 35 subjects who already had dementia at baseline, 111 subjects whose dietary questionnaires were not available, 1 subject with no blood sample, and 10 subjects who had implausible total energy intake (more or less than the mean energy intake ±3 standard deviations [< 640 or > 3084 kcal/day for men and < 698 or > 2450 kcal/day for women]), the remaining 1071 subjects (452 men and 619 women) were enrolled in the present study (Fig. S1).

### Follow-up survey

The subjects were followed up prospectively for 24 years, from December 1988 to November 2012 [25, 26]. During this period, health examinations were performed every year. Health information was checked yearly by letter or telephone call for any subjects who did not have examinations or who had moved out of town. We created a daily monitoring system in the study group and local physicians or members of the Health and Welfare Office of the town to identify new events, including stroke, cognitive impairment, and dementia. Comprehensive screening surveys of cognitive function including neuropsychological tests, i.e., the Hasegawa Dementia Scale [27], the Hasegawa Dementia Scale-Revised [28], or the Mini-Mental State Examination [29], were conducted in 1992, 1998, 2005, and 2012 (participation rate > 90% in all surveys), in which the surveys at home visits and nursing home visit were also conducted [4, 25]. When a subject was suspected of having new neurologic symptoms, including cognitive impairment, the subject was carefully evaluated by the study physician and psychiatrist. During the 24 years of follow-up, 759 (70.9%) subjects died. There were no subjects lost to follow-up, and no subjects for whom mortality status was unknown in this cohort.

### Diagnosis of dementia

Dementia was diagnosed according to the guidelines of the Diagnostic and Statistical Manual of Mental Disorders, Revised Third Edition (DSM-III–R) [30]. Subjects diagnosed with AD upon meeting the criteria of the National Institute of Neurological and Communicative Disorders and Stroke and the Alzheimer’s Disease and Related Disorders Association [31], and subjects diagnosed with vascular dementia (VaD) upon meeting the criteria of the National Institute of Neurological Disorders and Stroke and the Association Internationale pour la Recherche et l’Enseignement en Neurosciences were used to determine the diagnoses of VaD [32]. In addition, when a subject died, we collected and fully examined all the available medical information, including neuroimaging results (CT/MRI), performed a brain autopsy, and interviewed the family and attending physician of the deceased. The diagnostic procedure was reported previously [26].

During the follow-up period, a total of 464 subjects (151 men and 313 women) developed dementia, of which 286 had AD (236 had pure AD and 50 had AD with other coexisting subtypes) and 144 had VaD (100 had pure VaD and 44 had VaD with other coexisting subtypes). Thirty-three cases had mixed AD and VaD. Possible or probable dementia subtypes were diagnosed with clinical information including neuroimaging. Definite dementia subtypes were also determined on the basis of clinical and neuropathologic information [33, 34]. The cases of every subject suspected of having dementia and every subject who died during the follow-up period were adjudicated based on all available medical information by expert psychiatrists and expert stroke physicians to confirm the absence or presence of dementia and its subtypes. Among the subjects with dementia, 413 (89.0%) were evaluated using brain imaging, and 240 (51.7%) underwent autopsy; both were performed in 218 subjects (47.0%). Thus, 435 subjects in all (93.8%) underwent some kind of morphological examination. These cases were counted as events in the analyses for each subtype.

### Dietary survey

At our baseline screening examination in 1988, only one dietary survey was conducted using a 70-item semiquantitative food frequency questionnaire concerning food intake; the questionnaire was written in Japanese [35]. In the present study, vegetables consisted of green and yellow vegetables (e.g. spinach, tomato, pepper, carrot), other vegetables (e.g. cabbage, onion, cucumber) and pickles. The validity of this questionnaire was reported previously [36]. Trained dietitians interviewed participants to obtain their food intake, which consisted of quantitative information on individual foods consumed during the past month [37]. The questionnaire was completed by each subject before the examination and was checked by trained dietitians during the examination. The correlation coefficients between the food frequency questionnaire and dietary records were 0.58 for green and yellow vegetables, 0.33 for other vegetables, 0.55 for pickles, and 0.40 for fruits. The average food intake per day was calculated from the weekly frequency of intake of a diverse range of foods and the size of each portion. Nutritional intake was calculated from the food intake based on the 4th Revision of the Standard Tables of Food Composition in Japan [38]. The density method was used to adjust all dietary nutrients for total energy [39].

### Risk-factor measurements

At baseline, each subject completed a self-administered questionnaire covering medical history, antidiabetic and antihypertensive medications, educational level, alcohol consumption, smoking habits, and physical activity. History of stroke was defined as a pre-existing stroke event that consisted of a sudden onset of nonconvulsive and focal neurologic deficits that persisted > 24 h due to ischemia or hemorrhage, on the bases of all available clinical data, including medical records, neurological examination, and brain imaging. A low educational level was defined as ≤6 years of formal education. Smoking and drinking habits were classified as currently used or not. Regular exercise was defined as engaging in sports or other forms of exertion ≥3 times a week during leisure time. Blood pressure was measured three times using a standard mercury sphygmomanometer in the sitting position after resting for at least 5 min. The mean of three measurements was used for the analysis. Hypertension was defined as blood pressure of ≥140/90 mmHg or current use of antihypertensive drugs [40]. Electrocardiogram abnormalities were defined by atrial fibrillation (Minnesota Code = 8-3), ST depression (4-1, 2, 3), or left ventricular hypertrophy (3-1) [41]. Diabetes was defined mainly by a 75-g oral glucose tolerance test according to 1998 World Health Organization criteria [42], in addition to a medical history or treatment of diabetes. Serum total cholesterol concentrations were measured by the enzymatic method. Hypercholesterolemia was defined as total cholesterol ≥220 mg/dL [43]. Body height and weight were measured in light clothing without shoes, and body mass index (BMI) (kg/m^2^) was calculated.

### Statistical analysis

Intakes of vegetables, fruits, dietary fibers, vitamins, and minerals were divided into four categories based on the sex-specific quartile distribution. The values of the dietary intakes of milk and dairy products, and alcoholic beverages were natural log transformed for analysis, because of the skew of the distributions of these variables. *P*-values for the differences in the risk factors between the groups were calculated from linear and logistic regressions by cording the quartiles (Q1 as a reference). The incidence of dementia was calculated by the person-years method. The age- and sex-adjusted incidence rates of total dementia were calculated using a Poisson regression model including age and sex, where the variables of log-transformed person-years were used as the offset term [44]. The Cox proportional hazards model was used to estimate the adjusted hazard ratios (HRs) with the 95% confidence intervals (CIs) of total dementia, AD, and VaD according to the intakes of vegetables, fruits, dietary fibers, vitamins, and minerals. The confounding variables considered were age, sex, educational level, history of stroke, diabetes, systolic blood pressure, use of antihypertensive agents, electrocardiogram abnormalities, serum total cholesterol, BMI, current drinking, current smoking, regular exercise, and intakes of total energy, protein, fat, and carbohydrate. There were no variables with missing values, except for educational level. Subjects with missing values on educational level (*n* = 11, 1.0% of the study subjects) were excluded from the multivariable analysis for each relevant variable. The linear trends in the risk estimates were tested using the Cox model including the intake levels of each food, and nutrients with ordinal numbers (0, 1, 2, and 3) and the relevant covariates. The test of interaction was performed by adding a multiplicative interaction terms to the relevant Cox model. We also used restricted cubic splines to show the shape of these associations with 5 knots placed at the 5th, 25th, 50th, 75th and 95th percentiles of vegetable intake (107, 197, 281, 362 and 530 g/day, respectively) and fruit intake (0, 32, 75, 107, and 214 g/day, respectively) [45]. Our choice of the number of knots was based on the comparisons of model fit statistics (the Akaike information criterion) [46]. The 5th percentile was chosen as the reference value. To examine whether there was a need for cubic spline terms in addition to a linear term, the non-linearity for the association was tested by using a likelihood ratio test comparing the relevant model with only a linear term against the model with linear and cubic spline terms [45]. Because the *P* values for non-linearity of the association of vegetable intakes and fruit intakes with the risk of dementia were 0.11 and 0.03, respectively, we decided to add the cubic spline term to the relevant model when examining the association between continuous variables of vegetable and fruit intakes and dementia risk. The Spearman’s correlation coefficients were used for estimating the correlation of vegetables or fruits with the intakes of individual nutrients and foods. We considered correlation coefficients of 0.30 to 0.49 (or − 0.49 to − 0.30), 0.50 to 0.69 (or − 0.69 to − 0.50), ≥0.70 (or ≤ − 0.70) as weak, moderate, and strong correlations, respectively [47]. All statistical analyses were performed with the SAS 9.4 software (SAS Institute, Cary, NC). In all analyses, two-sided *p*-values (*P* < 0.05) were used for judging the statistical significance.

## Results

The baseline characteristics of subjects according to vegetable and fruit intake levels are summarized in Table 1. Compared to the group with the lowest vegetable intake, the proportions of current smoker in the third quartile and participants who engaging regular exercise in the second quartile was smaller, while proportion of regular exerciser in the third quartile was greater. The mean value of protein intake was significantly higher in the second- to the fourth quartile of vegetable intake than the first quartile, and fat intake was significantly higher in the second quartile, whereas that of total energy intake was lower in the highest quartile of vegetable intake, and systolic blood pressure and carbohydrate intake was significantly lower in the second quartile. With regard to fruit intake, the mean values of BMI and carbohydrate intake was higher in the highest quartile of fruit intake than the lowest quartile. The proportions of participants with low educational level and alcohol drinking was smaller in the highest quartile of fruit intake compared to the lowest quartile. The proportions of current smoker were significantly lower in the second- to the fourth quartile. Mean fat intake was significantly higher in the second quartile than the first quartile, while the third and the fourth quartile was not.

**Table 1:** Baseline characteristics of potential risk factors for dementia according to quartiles of vegetable and fruit intake^a^

Characteristic	Vegetable intake (g/1000 kcal)				Fruit intake (g/1000 kcal)			
	Q1	Q2	Q3	Q4	Q1	Q2	Q3	Q4
	M: ≤111 W: ≤135 (*n* = 267)	M: 112-151 W: 136-189 (*n* = 268)	M: 152-205 W: 190-250 (*n* = 268)	M: ≥206 W: ≥251 (*n* = 268)	M: ≤13 W: ≤22 (*n* = 267)	M: 14-30 W: 23-46 (*n* = 268)	M: 31-56 W: 47-71 (*n* = 268)	M: ≥57 W: ≥72 (*n* = 268)
Vegetable or fruit intake (median)								
Men (g/1000 kcal)	82	134	177	250	4	21	40	88
Women (g/1000 kcal)	110	163	217	292	12	34	54	107
***Clinical parameters***								
Age, y	69.3 (6.3)^b^	68.9 (6.6)	69.9 (6.6)	69.7 (6.5)	69.3 (6.8)	69.2 (6.7)	69.9 (6.5)	68.4 (6.1)
Men, %	42.3	42.2	42.2	42.2	42.3	42.2	42.2	42.2
Education ≤6 years, % ^c^	14.1	14.7	10.6	10.8	14.8	13.3	14.3	7.8^**^
History of stroke, %	4.5	3.0	5.6	4.5	5.6	4.5	3.7	3.7
Diabetes mellitus, %	11.6	15.3	17.2	14.9	15.0	14.2	13.1	16.8
Body mass index, kg/m^2^	22.2 (3.0)	22.0 (3.2)	22.4 (3.0)	22.6 (3.1)	22.0 (3.1)	22.3 (2.9)	22.3 (3.2)	22.6^*^ (3.1)
Systolic blood pressure, mmHg	140 (23)	136 (21)^*^	140 (23)	140 (23)	139 (21)	138 (23)	139 (23)	139 (22)
Diastolic blood pressure, mmHg	76 (11)	75 (10)	76 (10)	76 (11)	76 (11)	76 (11)	75 (10)	76 (11)
Antihypertensive agents, %	22.9	24.6	26.5	25.0	23.6	24.3	23.1	28.0
Electrocardiogram abnormalities, %	22.1	16.4	20.5	22.0	24.0	19.0	17.9	20.2
Serum total cholesterol, mg/dL	209 (43)	210 (48)	209 (44)	208 (42)	204 (43)	208 (48)	213 (43) ^*^	211 (42)
Current smoking, %	25.5	26.1	18.3^*^	22.8	30.7	27.2^*^	16.4^**^	18.3^*^
Current alcohol drinking, %	28.1	25.8	29.1	21.6	36.3	25.0	23.5	19.8^**^
Regular exercise, %	12.7	9.7^**^	19.4^*^	17.5	13.5	15.7	16.4	13.8
***Dietary factors***								
Total energy, kcal/day	1644 (416)	1653 (369)	1661 (390)	1531 (320) ^**^	1611 (413)	1628 (409)	1621 (350)	1628 (340)
Protein, g/1000 kcal	31.4 (5.3)	33.7 (5.6) ^**^	33.3 (5.2) ^**^	34.2 (6.0) ^**^	32.7 (6.0)	33.3 (6.1)	33.2 (5.1)	33.4 (5.2)
Fat, g/1000 kcal	28.4 (5.9)	29.5 (6.0) ^*^	28.7 (6.4)	28.5 (6.3)	28.2 (6.7)	29.6 (6.7) ^**^	28.6 (5.6)	28.7 (5.5)
Carbohydrate, g/1000 kcal	140.7 (18.5)	136.9 (18.7) ^*^	138.9 (20.1)	141.1 (18.2)	137.9 (20.7)	136.8 (19.7)	139.9 (17.9)	142.9 (16.9) ^**^

^a^ *M* men, *W* women, *Q* quartile

^b^ Values are expressed as the mean (standard deviation) or frequency

^c^ *n* = 1060

^*^P-values for the differences in the risk factors between groups were calculated from linear and logistic regressions by cording the quartiles as dummy variables (Q1 as a reference, *P < 0.05, **P < 0.01)

The baseline amounts of other nutrients and foods are also presented by quartiles of vegetable and fruit consumption in Table S1 and Table S2. In addition, vegetable intake was moderately or strongly correlated with intakes of several nutrients including total and insoluble dietary fibers, vitamin A, thiamin, vitamin C, potassium, and magnesium (all correlation coefficients ≥0.50). Likewise, fruit intake was also correlated with these nutrients, except for vitamin A (all correlation coefficients ≥0.30) (Table S3).

**Fig. 1:**
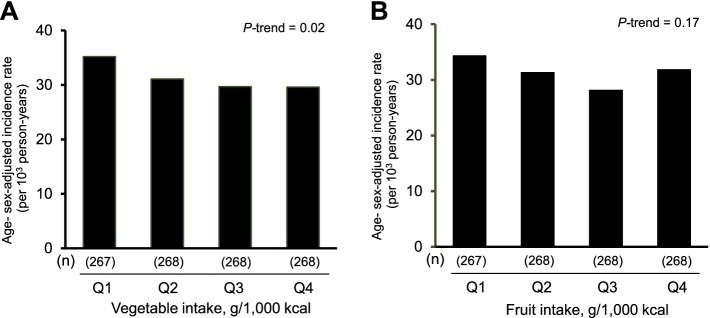
Age- and sex-adjusted incidence of total dementia according to quartiles of vegetable (**A**) and fruit intake (**B**) at baseline in older Japanese adults aged ≥60 years from 1988 to 2012. The incidence of dementia was calculated by the person-years method. The age- and sex-adjusted incidence rates of total dementia were calculated using a Poisson regression model including age and sex, where the variables of log-transformed person-years were used as the offset term

**Fig. 2:**
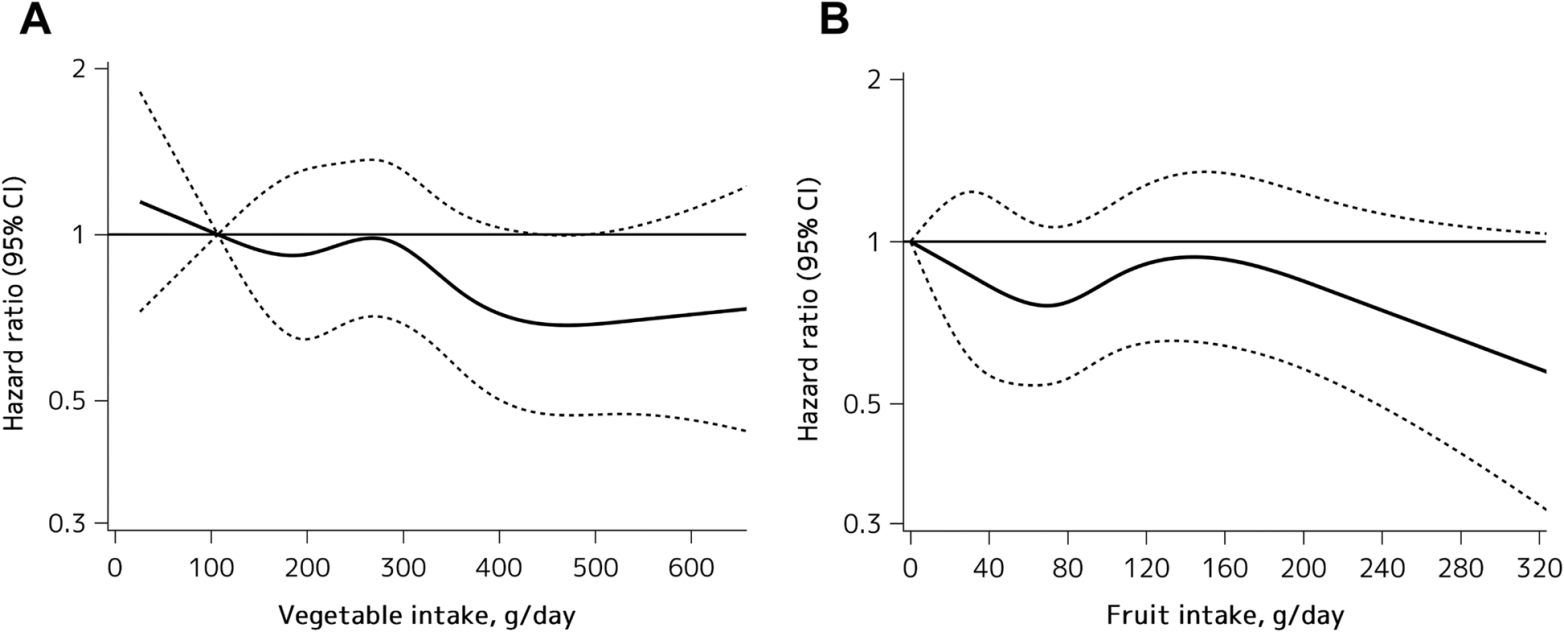
Restricted cubic splines for the association of vegetable intake (**A**) and fruit intake (**B**) with the risk of total dementia. Solid lines represent the hazard ratio, and dashed lines represent the 95% Cl of the hazard ratio. Knots were placed at the 5th, 25th, 50th, 75th, and 95th percentiles (107, 197, 281, 362, and 530 g/day) of vegetable intake, and at the same percentiles (0, 32, 75, 107, and 214 g/day) of fruit inake. A reference point was set at the 5th percentile (107 g/day for vegetable intake and 0 g/day for fruit intake). The X-axis in each graph shows up to the 99th percentile vaule of vegtable intake (682 g/day) or fruit intake (325 g/day). The risk estimates were adjusted for age, sex, educational level, history of stroke, diabetes, systolic blood pressure, use of antihypertensive agents, electrocardiogram abnormalities, total cholesterol, body mass index, current drinking, current smoking, regular exercise, and intakes of total energy, protein, fat, and carbohydrate

The age- and sex-adjusted incidence rate of total dementia decreased significantly with increasing vegetable intake levels (*P*-trend = 0.02), whereas there was no significant association between the fruit intake and the incidence rate of total dementia (*P*-trend = 0.17) (Fig. 1). As shown in Table 2, the age- and sex-adjusted HR of the development of total dementia decreased significantly with increasing vegetable intake levels (*P*-trend = 0.02). This downward association remained significant after adjusting for age, sex, educational level, history of stroke, diabetes, systolic blood pressure, use of antihypertensive agents, electrocardiogram abnormalities, serum total cholesterol, BMI, current drinking, current smoking, regular exercise, and intakes of total energy, protein, fat, and carbohydrate (*P*-trend = 0.03). For total dementia, the multivariable-adjusted HR was significantly lower in subjects at the highest quartile of vegetable intake than in those at the lowest quartile (HR 0.73; 95% CI 0.56, 0.96). There was no evidence of interaction in the association between vegetable intakes and the risk of total dementia between the subgroups of age, sex, education levels and current smoking status (all p for interaction > 0.31) (Table S4). With regard to dementia subtypes, the multivariable-adjusted HR of AD was significantly lower in subjects at the highest quartile of vegetable intake than in those at the lowest quartile (HR 0.69; 95% CI 0.49, 0.98; *P*-trend = 0.049), but no significant association was observed for VaD (*P*-trend = 0.24) (Table 3). With regard to fruit intake, compared to the first quartile, the multivariable-adjusted HR of total dementia was significantly lower in the third quartile of fruit intake (adjusted HR 0.76; 95% CI: 0.58, 0.99; *P* = 0.04), but there was no significant association of the fruit intake with the risk of total dementia (*P*-trend = 0.31) (Table 2). The multivariable-adjusted HR of VaD decreased with higher fruit intake, but this association was not significant (*P*-trend = 0.07), and there was no significant association between fruit intake and AD (*P*-trend = 0.56). (Table 3). As for the combined intakes of vegetables and fruits, higher intakes of vegetables and fruits were significantly associated with lower risk of total dementia and VaD (Table S5 and Table S6). As a sensitivity analysis, the association between vegetable and fruit intake and dementia was examined by excluding those who developed dementia within a follow-up period of 2 years, but the findings did not change substantially (Table S7 and Table S8). Moreover, since the recommended amount of vegetable intake is generally shown as an absolute value, we performed an additional analysis by using the absolute values of vegetable and fruit intake. The baseline characteristics according to the quartiles of the absolute values of vegetable or fruit intake are shown in Table S9. The results showed that higher absolute values of the vegetable intake were significantly associated with lower risks of total dementia and AD after adjusting for potential confounding factors (Table S10 and Table S11, and Fig. S2). We also addressed the association of intakes of vegetables and fruits with dementia risk by using a restricted cubic spline model. As shown in Fig. 2, the multivariable-adjusted HRs of incident dementia tended to decrease with higher amounts of vegetable intakes from around 270 g/day, and the upper limits of the 95% confidence interval were < 1.0 at around 400 g/day against the reference value (=107 g/day for vegetable intake). On the other hand, there was no clear association between the intakes of fruit and the risk of dementia. In Japan, the recommended amounts are ≥350 g/day for vegetable intake and ≥ 200 g/day for fruit intake [48]. Therefore, we also compared the risk of total dementia between subjects with intakes above and below these recommended amounts. Participants with vegetable intake of ≥350 g/day had a significantly lower risk of total dementia than those with an intake < 350 g/day after adjusting for the aforementioned confounders (HR 0.76; 95% CI 0.60, 0.95), whereas there was no evidence of significant difference in the dementia risk between subjects with fruit intake of ≥200 g/day and those with fruit intake of < 200 g/day (HR 0.83; 95% CI 0.55, 1.24).

**Table 2:** Adjusted hazard ratios of total dementia according to quartiles of vegetable and fruit intake^a^

Food intake levels (g/1000 kcal)	Number of events/ PYs	Hazard ratio (95% CI)	
		Age- and sex-adjusted	Multivariable-adjusted ^b, c^
**Vegetable**			
Q1 (M: ≤111; W: ≤135)	120/3576	1.00 (reference)	1.00 (reference)
Q2 (M: 112-151; W: 136-189)	117/3841	0.82 (0.63, 1.05)	0.84 (0.65, 1.09)
Q3 (M: 152-205; W: 190-250)	111/3754	0.80 (0.62, 1.03)	0.85 (0.65, 1.11)
Q4 (M: ≥206; W: ≥251)	116/3981	0.73 (0.56, 0.94)	0.73 (0.56, 0.96)
*P-*trend		0.02	0.03
**Fruit**			
Q1 (M: ≤13; W: ≤22)	118/3579	1.00 (reference)	1.00 (reference)
Q2 (M: 14-30; W: 23-46)	116/3801	0.84 (0.65, 1.09)	0.87 (0.67, 1.13)
Q3 (M: 31-56; W: 47-71)	108/3885	0.74 (0.57, 0.96)	0.76 (0.58, 0.99)
Q4 (M:≥ 57; W: ≥72)	122/3887	0.86 (0.67, 1.11)	0.90 (0.69, 1.17)
*P-*trend		0.17	0.31

^a^ *M* men, *W* women, *PYs* person-years, *Q* quartile

^b^ Adjusted for age, sex, educational level, history of stroke, diabetes, systolic blood pressure, use of antihypertensive agents, electrocardiogram abnormalities, total cholesterol, body mass index, current drinking, current smoking, regular exercise, and intakes of total energy, protein, fat, and carbohydrate

^c^ *n* = 1060

**Table 3:** Adjusted hazard ratios of Alzheimer’s disease and vascular dementia according to quartiles of vegetable and fruit intake^a^

Food intake levels (g/1000 kcal)	Alzheimer’s disease				Vascular dementia			
	Number of events/ PYs	Age- and sex-adjusted incidence (per 10^3^ PYs)	Hazard ratio (95% CI)		Number of events/ PYs	Age- and sex-adjusted incidence (per 10^3^ PYs)	Hazard ratio (95% CI)	
			Age- and sex-adjusted	Multivariable-adjusted^b,c^			Age- and sex-adjusted	Multivariable-adjusted ^b, c^
**Vegetable**								
Q1 (M: ≤111; W: ≤135)	75/3576	20.9	1.00 (reference)	1.00 (reference)	35/3576	10.3	1.00 (reference)	1.00 (reference)
Q2 (M: 112-151; W: 136-189)	72/3841	18.0	0.77 (0.55, 1.06)	0.75 (0.54, 1.05)	40/3841	10.9	1.01 (0.64, 1.59)	1.16 (0.73, 1.84)
Q3 (M: 152-205; W: 190-250)	64/3754	16.2	0.72 (0.52, 1.01)	0.73 (0.52, 1.03)	37/3754	10.1	0.94 (0.59, 1.49)	1.16 (0.72, 1.86)
Q4 (M: ≥206; W: ≥251)	75/3981	18.0	0.71 (0.52, 0.98)	0.69 (0.49, 0.98)	32/3981	8.3	0.75 (0.46, 1.21)	0.73 (0.44, 1.21)
*P-*trend			0.045	0.049			0.21	0.24
**Fruit**								
Q1 (M: ≤13; W: ≤22)	69/3579	19.0	1.00 (reference)	1.00 (reference)	44/3579	13.0	1.00 (reference)	1.00 (reference)
Q2 (M: 14-30; W: 23-46)	59/3801	14.8	0.71 (0.50, 1.01)	0.73 (0.51, 1.04)	38/3801	10.6	0.77 (0.50, 1.19)	0.79 (0.51, 1.24)
Q3 (M: 31-56; W: 47-71)	76/3885	18.7	0.90 (0.65, 1.24)	0.88 (0.63, 1.23)	29/3885	7.6	0.53 (0.33, 0.85)	0.63 (0.39, 1.02)
Q4 (M: ≥57; W: ≥72)	82/3887	20.3	1.00 (0.73, 1.38)	1.03 (0.74, 1.44)	33/3887	8.7	0.62 (0.39, 0.97)	0.68 (0.43, 1.09)
*P-*trend			0.59	0.56			0.01	0.07

^a^ *M* men, *W* women, *PYs* person-years, *Q* quartile

^b^ Adjusted for age, sex, educational level, history of stroke, diabetes, systolic blood pressure, use of antihypertensive agent, electrocardiogram abnormalities, total cholesterol, body mass index, current drinking, current smoking, regular exercise, and intakes of total energy, protein, fat, and carbohydrate

^c^ *n* = 1060

Next, we investigated the association of the intakes of individual nutrients of vegetable and fruit with the risk of dementia. As shown in Table 4, the multivariable-adjusted HR of total dementia and AD showed a tendency to decrease with higher intakes of total dietary fiber and insoluble dietary fiber, but not with higher intake of soluble dietary fiber, and none of these associations reached the level of statistical significance (all *P*-trend < 0.1). There was no clear evidence to suggest a link between intakes of total dietary fiber, soluble fiber, or insoluble dietary fiber and the risk of VaD (all *P*-trend > 0.30). Next, we examined the associations of vitamin and mineral intake levels with the development of total dementia and its subtypes (Table 5). The multivariable-adjusted HR of total dementia was significantly lower for subjects with higher intakes of vitamin A, riboflavin, vitamin C, magnesium, calcium, and potassium (all *P*-trend < 0.05). With regard to subtypes of dementia, the multivariable-adjusted HR of AD was significantly lower for subjects with higher intakes of riboflavin (*P*-trend = 0.04), while the multivariable-adjusted HRs of VaD were significantly lower for subjects with higher intakes of vitamin A, riboflavin, vitamin C and calcium (all *P*-trend < 0.05).

**Table 4:** Adjusted hazard ratios of total dementia, Alzheimer’s disease, and vascular dementia according to quartiles of dietary fiber intake^a^

Nutrient intake levels (g/1000 kcal)	Total dementia		Alzheimer’s disease		Vascular dementia	
	Number of events/ PYs	Hazard ratio (95% CI)	Number of events/ PYs	Hazard ratio (95% CI)	Number of events/ PYs	Hazard ratio (95% CI)
		Multivariable-adjusted^b,c^		Multivariable-adjusted^b,c^		Multivariable-adjusted^b,c^
**Total dietary fiber**						
Q1 (M: ≤4.74; W: ≤5.79)	114/3455	1.00 (reference)	75/3455	1.00 (reference)	31/3455	1.00 (reference)
Q2 (M: 4.75-5.95; W: 5.80-7.14)	125/3857	0.92 (0.71, 1.18)	75/3857	0.85 (0.61, 1.17)	40/3857	1.06 (0.66, 1.71)
Q3 (M: 5.96-7.40; W: 7.15-8.49)	107/3925	0.75 (0.57, 0.99)	64/3925	0.70 (0.49, 0.99)	37/3925	0.94 (0.57, 1.55)
Q4 (M: ≥7.41; W: ≥8.50)	118/3914	0.79 (0.58, 1.08)	72/3914	0.74 (0.50, 1.11)	36/3914	0.90 (0.51, 1.59)
*P-*trend		0.07		0.08		0.65
**Soluble dietary fiber**						
Q1 (M: ≤0.65; W: ≤0.85)	114/3437	1.00 (reference)	70/3437	1.00 (reference)	34/3437	1.00 (reference)
Q2 (M: 0.66-1.05; W: 0.86-1.22)	122/3771	0.95 (0.73, 1.23)	74/3771	0.97 (0.70, 1.36)	45/3771	1.04 (0.66, 1.65)
Q3 (M: 1.06-1.47; W: 1.23-1.71)	110/4037	0.76 (0.57, 1.02)	73/4037	0.87 (0.60, 1.25)	26/4037	0.55 (0.31, 0.97)
Q4 (M: ≥1.48; W: ≥1.72)	118/3906	0.86 (0.62, 1.20)	69/3906	0.87 (0.57, 1.34)	39/3906	0.84 (0.47, 1.51)
*P-*trend		0.20		0.44		0.30
**Insoluble dietary fiber**						
Q1 (M: ≤4.02; W: ≤4.83)	118/3471	1.00 (reference)	77/3471	1.00 (reference)	33/3471	1.00 (reference)
Q2 (M: 4.03-4.91; W: 4.84-5.83)	114/3783	0.78 (0.60, 1.02)	68/3783	0.72 (0.52, 1.00)	35/3783	0.86 (0.53, 1.39)
Q3 (M: 4.92-5.95; W: 5.84-6.81)	116/3940	0.75 (0.58, 0.99)	71/3940	0.69 (0.49, 0.98)	42/3940	0.99 (0.61, 1.60)
Q4 (M: ≥5.96; W: ≥6.82)	116/3957	0.75 (0.56, 1.02)	70/3957	0.69 (0.47, 1.00)	34/3957	0.82 (0.47, 1.42)
*P-*trend		0.06		0.051		0.63

^a^ *M* men, *W* women, *PYs* person-years, *Q* quartile

^b^ Adjusted for age, sex, educational level, history of stroke, diabetes, systolic blood pressure, use of antihypertensive agent, electrocardiogram abnormalities, total cholesterol, body mass index, current drinking, current smoking, regular exercise, and intakes of total energy, protein, fat, and carbohydrate

^c^ *n* = 1060

**Table 5:** Adjusted hazard ratios of total dementia, Alzheimer’s disease, and vascular dementia according to quartiles of vitamin, and mineral intake^a^

Nutrient intake levels	Total dementia		Alzheimer’s disease		Vascular dementia	
	Number of events/ PYs	Hazard ratio (95% CI)	Number of events/ PYs	Hazard ratio (95% CI)	Number of events/ PYs	Hazard ratio (95% CI)
		Multivariable-adjusted^b,c^		Multivariable-adjusted^b,c^		Multivariable-adjusted^b,c^
**Vitamin A (I.U/1000 kcal)**						
Q1 (M: ≤1178; W: ≤1446)	113/3560	1.00 (reference)	69/3560	1.00 (reference)	37/3560	1.00 (reference)
Q2 (M: 1179-1575; W: 1447-1884)	128/3870	0.91 (0.70, 1.18)	72/3870	0.84 (0.59, 1.17)	46/3870	1.01 (0.64, 1.59)
Q3 (M: 1576-2044; W: 1885-2371)	118/3756	0.99 (0.76, 1.29)	79/3757	1.06 (0.76, 1.48)	32/3757	0.86 (0.53, 1.40)
Q4 (M: ≥2045; W: ≥2372)	105/3964	0.69 (0.52, 0.91)	66/3964	0.69 (0.49, 0.99)	29/3964	0.57 (0.34, 0.95)
*P-*trend		0.02		0.13		0.02
**Thiamin (mg/1000 kcal)**						
Q1 (M: ≤0.36; W: ≤0.39)	122/3586	1.00 (reference)	78/3586	1.00 (reference)	36/3586	1.00 (reference)
Q2 (M: 0.37-0.40; W: 0.40-0.44)	126/3940	0.82 (0.63, 1.06)	70/3940	0.69 (0.49, 0.96)	49/3940	1.14 (0.72, 1.81)
Q3 (M: 0.41-0.46; W: 0.45-0.49)	111/3851	0.71 (0.54, 0.95)	70/3851	0.67 (0.47, 0.95)	34/3851	0.80 (0.48, 1.35)
Q4 (M: ≥0.47; W: ≥0.50)	105/3774	0.77 (0.58, 1.03)	68/3774	0.77 (0.53, 1.10)	25/3774	0.64 (0.36, 1.12)
*P-*trend		0.06		0.19		0.05
**Riboflavin (mg/1000 kcal)**						
Q1 (M: ≤0.52; W: ≤0.58)	114/3435	1.00 (reference)	70/3435	1.00 (reference)	42/3435	1.00 (reference)
Q2 (M: 0.53-0.64; W: 0.59-0.71)	123/3837	0.94 (0.71, 1.24)	80/3837	0.97 (0.68, 1.38)	31/3837	0.63 (0.38, 1.05)
Q3 (M: 0.65-0.77; W: 0.72-0.84)	112/3963	0.70 (0.50, 0.98)	66/3963	0.68 (0.45, 1.05)	39/3963	0.57 (0.33, 1.00)
Q4 (M: ≥0.78; W: ≥0.85)	115/3916	0.61 (0.39, 0.95)	70/3916	0.61 (0.34, 1.07)	32/3916	0.38 (0.17, 0.83)
*P-*trend		0.01		0.04		0.02
**Vitamin C (mg/1000 kcal)**						
Q1 (M: ≤30; W: ≤38)	115/3516	1.00 (reference)	78/3516	1.00 (reference)	31/3516	1.00 (reference)
Q2 (M: 31-41; W: 39-50)	125/3892	0.92 (0.71, 1.19)	65/3892	0.69 (0.50, 0.97)	47/3892	1.37 (0.86, 2.18)
Q3 (M: 42-55; W: 51-66)	112/3723	0.83 (0.63, 1.08)	69/3723	0.73 (0.53, 1.02)	40/3723	1.15 (0.71, 1.86)
Q4 (M: ≥56; W:≥67)	112/4021	0.74 (0.56, 0.96)	74/4021	0.73 (0.52, 1.01)	26/4021	0.61 (0.36, 1.05)
*P-*trend		0.02		0.09		0.047
**Magnesium (mg/1000 kcal)**						
Q1 (M: ≤85; W: ≤94)	121/3491	1.00 (reference)	69/3491	1.00 (reference)	41/3491	1.00 (reference)
Q2 (M: 86-98; W: 95-107)	113/3759	0.77 (0.58, 1.01)	75/3759	0.84 (0.59, 1.19)	33/3759	0.78 (0.48, 1.26)
Q3 (M: 99-111; W: 108-124)	113/3999	0.61 (0.46, 0.83)	67/3999	0.63 (0.43, 0.93)	37/3999	0.64 (0.38, 1.07)
Q4 (M: ≥112; W: ≥125)	117/3902	0.69 (0.50, 0.95)	75/3902	0.79 (0.52, 1.19)	33/3902	0.63 (0.35, 1.13)
*P-*trend		0.02		0.17		0.10
**Calcium (mg/1000 kcal)**						
Q1 (M: ≤251; W: ≤263)	121/3507	1.00 (reference)	70/3507	1.00 (reference)	44/3507	1.00 (reference)
Q2 (M: 252-305; W: 264-328)	125/3838	0.81 (0.62, 1.06)	78/3838	0.95 (0.67, 1.34)	35/3838	0.63 (0.39, 1.02)
Q3 (M: 306-382; W: 329-394)	109/3897	0.59 (0.43, 0.80)	67/3897	0.69 (0.46, 1.03)	32/3897	0.50 (0.29, 0.87)
Q4 (M: ≥383; W: ≥395)	109/3910	0.50 (0.33, 0.74)	71/3910	0.70 (0.42, 1.19)	33/3910	0.39 (0.19, 0.78)
*P-*trend		< 0.001		0.08		0.01
**Potassium (mg/1000 kcal)**						
Q1 (M: ≤1060; W: ≤1200)	118/3551	1.00 (reference)	72/3551	1.00 (reference)	38/3551	1.00 (reference)
Q2 (M: 1061-1234; W: 1201-1396)	121/3723	0.86 (0.66, 1.13)	70/3723	0.82 (0.58, 1.16)	39/3723	0.95 (0.59, 1.53)
Q3 (M: 1235-1468; W: 1397-1659)	115/4058	0.71 (0.54, 0.95)	76/4058	0.77 (0.53, 1.10)	38/4058	0.80 (0.49, 1.31)
Q4 (M: ≥1469; W: ≥1660)	110/3819	0.74 (0.54, 1.02)	68/3819	0.79 (0.53, 1.18)	29/3819	0.58 (0.32, 1.05)
*P-*trend		0.03		0.24		0.06

^a^ *M* Men, *W* Women, *PYs* person-years, *Q* Quartile

^b^ Adjusted for age, sex, educational level, history of stroke, diabetes, systolic blood pressure, use of antihypertensive agent, electrocardiogram abnormalities, total cholesterol, body mass index, current drinking, current smoking, regular exercise, and intakes of total energy, protein, fat, and carbohydrate

^c^ *n* = 1060

## Discussion

In this 24-year follow-up study, we demonstrated that higher vegetable intake was associated significantly with a lower risk of total dementia in the Japanese older adults. The risk of dementia tended to decrease continuously with higher vegetable intakes from around 270 g/day to 400 g/day. With regard to dementia subtypes, subjects with higher vegetable intake were at significantly lower risk of AD, but no such association was observed for VaD. In addition, there was no clear evidence to suggest an association between fruit intake and the incidence of total dementia and its subtypes. Moreover, the risk of dementia tended to be lower with higher intakes of dietary fiber, vitamins, and minerals. These findings may provide important information on the association of vegetables and vegetable-containing nutrients with the risk of dementia in Japanese.

Previous epidemiological studies have shown that higher intake of vegetables and fruits is associated with lower incidence of dementia [16–19]. Most epidemiological studies have examined the association of combined intakes of vegetables and fruits with dementia [16–18]. A previous analysis from the Three-City Study demonstrated that daily consumptions of fruits and vegetables were associated with a lower risk of total dementia [17]. Similarly, the Swedish Twins Study also reported that moderate or high intake of vegetables and fruits was associated with a lower risk of total dementia and AD, as compared with no or low intake [18]. In addition, the Kame Project Cohort found that Japanese-American subjects who drank vegetable and fruit juices ≥3 times per week had a 16% lower risk of developing AD than those who drank them < 1 time per week [16]. When combined vegetable and fruit intake, similar results were observed (Table S5 and Table S6). These results are in line with previous studies [16–18]. On the other hand, few studies have separately examined the association of vegetable intake versus fruit intake with dementia risk. In a French study, raw vegetables, raw fruits, and cooked vegetables and fruits were not associated with dementia or AD [49]. Moreover, a Chinese study showed that consumption of at least two servings of fruit per day was associated with a reduction in dementia risk, but the same study found no clear association for vegetable intake [21]. Unlike in these two studies, we found a clear association between vegetable intake, but not fruit intake, and dementia. The difference in results between these previous reports and our present study might have been related to differences in the study design, definitions of fruits and vegetables used to calculate their intake [50], and the analysis methods among the studies. The difference in intake between quartiles was smaller for fruits than for vegetables, which may explain why there was no statistically significant association between fruits and dementia.

The possible mechanisms underlying the significant inverse association between the vegetable intake and the risk of dementia and AD in the present study are unclear. Vegetables are rich in vitamins, minerals and dietary fiber [8], and in fact we observed that vegetables had a strong positive correlation with intakes of total and insoluble dietary fibers, vitamin A, thiamin, vitamin C, potassium, and magnesium (Table S3). It is known that oxidative stress [51] and inflammation [52] are involved in the onset and progression of dementia [53], while vitamin A, B vitamins, and vitamin C in vegetables have antioxidant and anti-inflammatory properties [9–11]. Dietary fibers enhance the production of short-chain fatty acids, which have been considered to inhibit amyloid formation, through fermentation and degradation by intestinal bacteria [13, 14]. In addition, higher glucose levels may be a risk factor for dementia [54], and dietary fiber delays the absorption of carbohydrates and adequately secretes insulin, which leads to reduced post-prandial blood glucose levels [13]. With regard to minerals, we have previously reported that high intakes of magnesium, calcium, and potassium were linked with a reduced risk of dementia [12]. Higher intakes of these minerals have been reported to be associated with lower risk of hypertension, which is a risk factor for vascular diseases including VaD [55], and they have also been reported to suppress free radical formation and platelet aggregation, and to improve dyslipidemia, neuronal secretion of neurotransmitters, and insulin sensitivity [56–59]. Taken together, these results suggest that vegetable intake may contribute to dementia risk reduction through the actions of these nutrients.

The strengths of the present study include its longitudinal population-based design and its perfect follow-up of study subjects, in which the development of dementia and its subtypes were diagnosed as accurately as possible based on neuropsychological tests, medical examinations by the study physician and psychiatrist, clinical records, brain imaging, and autopsy findings. In addition, because the analysis was conducted using sex-specific quartiles of vegetables and fruits, the effect of residual confounding due to differences in vegetable intake between men and women was minimized. However, several limitations in the present study should be noted. First, there is a possibility of misclassification of intakes of vegetables, fruits, and their nutrients, because the dietary survey was conducted by using a self-reported semiquantitative food frequency questionnaire. Additionally, dietary intake was assessed only once at baseline. Therefore, we could not consider dietary changes during follow-up. These limitations could have led to some degree of misclassification of vegetable and fruit intake, which in turn could have weakened the association found in the present study. Second, it was difficult to fully exclude the effects of residual confounders (other nutrients, eating behaviors, and health consciousness) on the association between vegetables, fruit intake and risk of dementia. It has been reported that people who consume various food combinations that have synergistic effects (i.e., combinations with a good balance between constituents within the food) [60, 61] and who practice good overall lifestyle habits have a low risk of dementia [62]. In general, healthy dietary patterns can be generally described as those that are rich in health-promoting foods, including plant-based foods, fresh vegetables and fruits, and soybean and soybean products [63]. Since a typical diet contains a mixture of various foods, the intakes of various foods are positively or negatively correlated with the intakes of others [64]. In our study, the increase of vegetable intake was associated with the lower intake of rice and the higher intake of soybean and soybean products (Table S1). Therefore, the observed inverse association between vegetable intake and dementia risk might have been partly due to individuals who consumed more vegetables having healthier dietary habits. Third, because individual nutrients are correlated with each other, it may be difficult to distinguish the discrete association of individual nutrients on the risk of dementia. Moreover, the associations of individual nutrients were not able to identify the derived food (i.e., vegetables or fruits) of nutrients because similar nutrients are included in vegetables and fruits and the subjects with high vegetable intake consumes fruits higher. Fourth, since the number of cases of dementia subtypes was not particularly large, we cannot rule out the possibility that the statistical power could have been insufficient to detect the true association for dementia subtypes. Finally, information on supplements was not available.

## Conclusions

A long-term follow-up study of general older adults in Japan showed that higher vegetable intake was significantly associated with a lower risk of dementia. A diet rich in vitamins, minerals and dietary fiber in the form of vegetables may be beneficial in reducing the risk of dementia in the elderly segment of Japanese adults aged ≥60 years. Further investigations are needed to clarify the association between vegetable intake and the incidence of dementia in Asian populations.

## Supplementary Information

**Additional file 1: Supplementary Figure 1.** Participant flow chart.

**Additional file 2: Figure S2.** Age- and sex-adjusted incidence of total dementia according to quartiles of the absolute values of the vegetable (A) and fruit intake (B) at baseline in older Japanese adults aged ≥60 years from 1988–2012.

**Additional file 3: Table S1.** Amounts of nutrients and foods according to quartiles of vegetable intake at baseline ^a^. **Table S2.** Amounts of nutrients and foods according to quartiles of fruit intake at baseline ^a^. **Table S3.** Spearman’s correlation coefficients between nutrients and intake of vegetables and fruits. **Table S4.** Multivariable-adjusted hazard ratios of total dementia per 1SD increment in vegetable intake between subgroups of age, sex, education levels and current smoking status in the study population, 1988-2012. **Table S5.** Adjusted hazard ratios of total dementia according to quartiles of the total consumption of the vegetable and fruit intake^a^. **Table S6.** Adjusted hazard ratios of Alzheimer's disease and vascular dementia according to quartiles of the total consumption of the vegetable and fruit intake ^a^. **Table S7.** Adjusted hazard ratios of total dementia according to quartiles of vegetable and fruit intake with the exclusion of subjects developing dementia within a follow-up period of 2 years or less^a^. **Table S8.** Adjusted hazard ratios of Alzheimer's disease and vascular dementia according to quartiles of vegetable and fruit intake with the exclusion of subjects developing dementia within a follow-up period of 2 years or less^a^. **Table S9.** Baseline characteristics of potential risk factors for dementia according to quartiles of the absolute values of the vegetable and fruit intake ^a^. **Table S10.** Adjusted hazard ratios of total dementia according to quartiles of the absolute values of the vegetable and fruit intake^a^. **Table S11.** Adjusted hazard ratios of Alzheimer's disease and vascular dementia according to quartiles of the absolute values of the vegetable and fruit intake ^a^.

## Data Availability

Data described in the manuscript, code book, and analytic code will not be made available because they contain confidential clinical and demographic data of the study participants. However, further information about the datasets is available on reasonable request for purposes of replicating procedures and results, with the permission of the principal investigator of the Hisayama Study (Toshiharu Ninomiya, contact e-mail: info_eph@hisayamalife.or.jp).

## Abbreviations

AD: Alzheimer’s disease
VaD: Vascular dementia
DSM-III–R: Diagnostic and Statistical Manual of Mental Disorders, Revised Third Edition
BMI: Body mass index
HRs: Hazard ratios
CIs: Confidence intervals
