# Supplementary material for: Long-term association of vegetable and fruit intake with risk of dementia in Japanese older adults: the Hisayama study
Source: BMC Geriatr. 2022 Mar 28;22:257. doi: 10.1186/s12877-022-02939-2 (PMC8962464; doi:10.1186/s12877-022-02939-2)
Supplement: Supplementary file 1 — Additional file 1: Supplementary Figure 1. Participant flow chart. [file 12877_2022_2939_MOESM1_ESM.pdf]

Online Supplementary Material

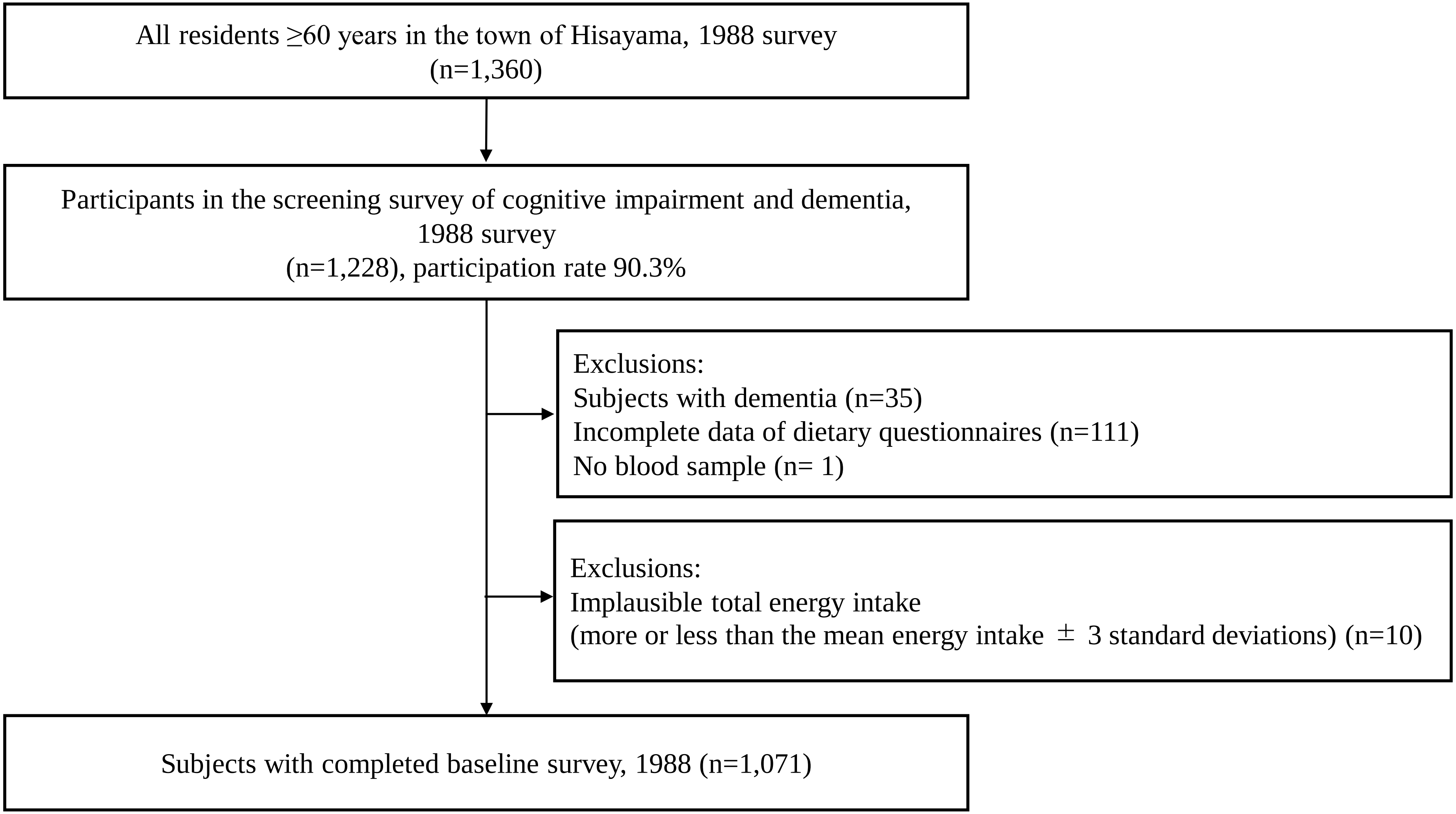

Supplementary Figure 1. Participant flow chart
