# Supplementary material for: Long-term association of vegetable and fruit intake with risk of dementia in Japanese older adults: the Hisayama study
Source: BMC Geriatr. 2022 Mar 28;22:257. doi: 10.1186/s12877-022-02939-2 (PMC8962464; doi:10.1186/s12877-022-02939-2)
Supplement: Supplementary file 2 — Additional file 2: Figure S2. Age- and sex-adjusted incidence of total dementia according to quartiles of the absolute values of the vegetable (A) and fruit intake (B) at baseline in older Japanese adults aged ≥60 years from 1988–2012. [file 12877_2022_2939_MOESM2_ESM.pptx]

## Slide 1
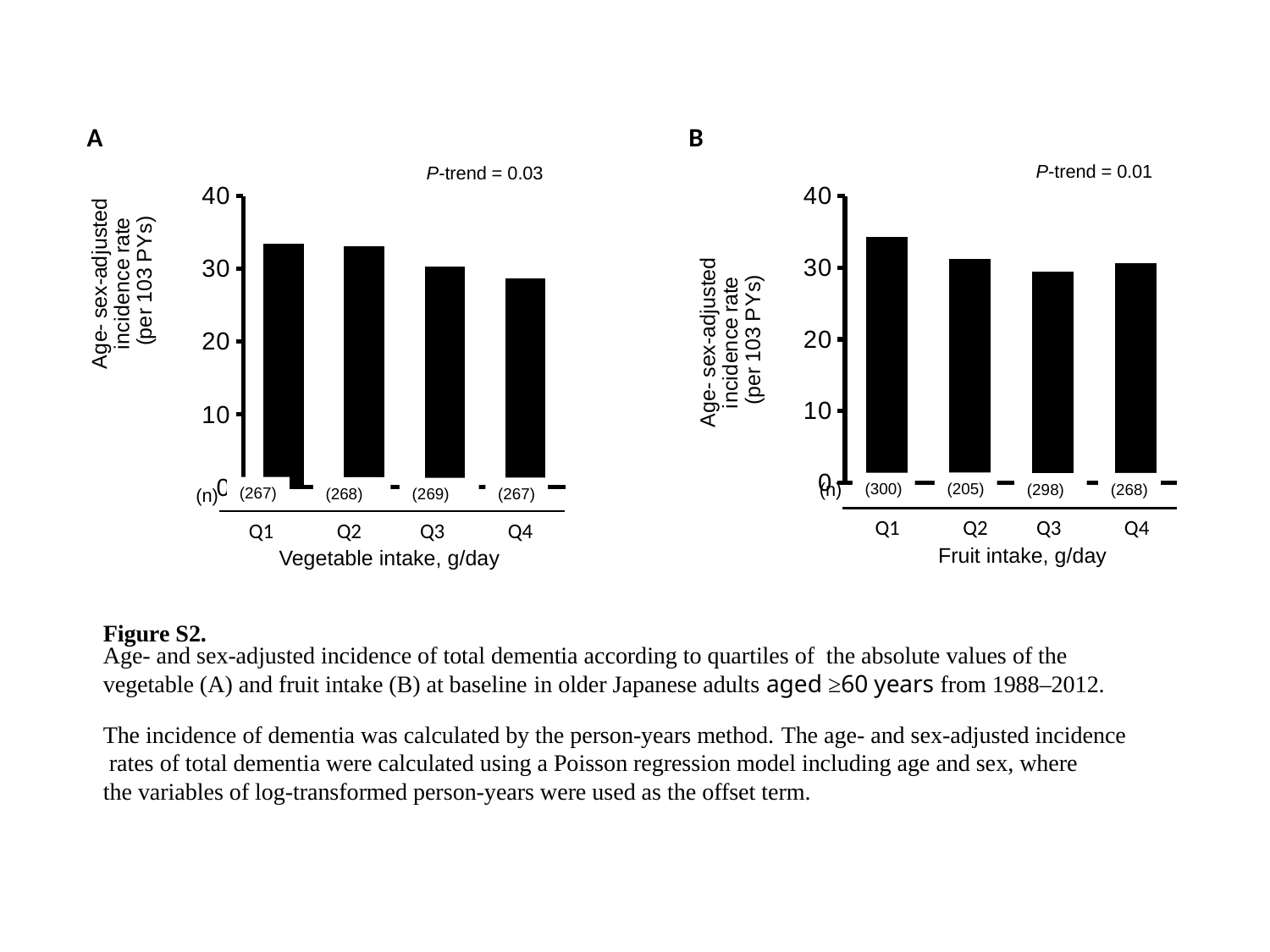

A
B
P-trend = 0.01
P-trend = 0.03
### Chart
| Category | |
|---|---|
### Chart
| Category | |
|---|---|(n)
(205)
(300)
(268)
(298)
(267)
(268)
(n)
(267)
(269)
Q1 Q2 Q3 Q4
Q1 Q2 Q3 Q4
Fruit intake, g/day
Vegetable intake, g/day
Figure S2.
Age- and sex-adjusted incidence of total dementia according to quartiles of the absolute values of the
vegetable (A) and fruit intake (B) at baseline in older Japanese adults aged ≥60 years from 1988–2012.
The incidence of dementia was calculated by the person-years method. The age- and sex-adjusted incidence
 rates of total dementia were calculated using a Poisson regression model including age and sex, where
the variables of log-transformed person-years were used as the offset term.
