# Supplementary material for: Long-term association of vegetable and fruit intake with risk of dementia in Japanese older adults: the Hisayama study
Source: BMC Geriatr. 2022 Mar 28;22:257. doi: 10.1186/s12877-022-02939-2 (PMC8962464; doi:10.1186/s12877-022-02939-2)
Supplement: Supplementary file 3 — Additional file 3: Table S1. Amounts of nutrients and foods according to quartiles of vegetable intake at baseline a. Table S2. Amounts of nutrients and foods according to quartiles of fruit intake at baseline a. Table S3. Spearman’s correlation coefficients between nutrients and intake of vegetables and fruits. Table S4. Multivariable-adjusted hazard ratios of total dementia per 1SD increment in vegetable intake between subgroups of age, sex, education levels and current smoking status in the study population, 1988-2012. Table S5. Adjusted hazard ratios of total dementia according to quartiles of the total consumption of the vegetable and fruit intakea. Table S6. Adjusted hazard ratios of Alzheimer's disease and vascular dementia according to quartiles of the total consumption of the vegetable and fruit intake a. Table S7. Adjusted hazard ratios of total dementia according to quartiles of vegetable and fruit intake with the exclusion of subjects developing dementia within a follow-up period of 2 years or lessa. Table S8. Adjusted hazard ratios of Alzheimer's disease and vascular dementia according to quartiles of vegetable and fruit intake with the exclusion of subjects developing dementia within a follow-up period of 2 years or lessa. Table S9. Baseline characteristics of potential risk factors for dementia according to quartiles of the absolute values of the vegetable and fruit intake a. Table S10. Adjusted hazard ratios of total dementia according to quartiles of the absolute values of the vegetable and fruit intakea. Table S11. Adjusted hazard ratios of Alzheimer's disease and vascular dementia according to quartiles of the absolute values of the vegetable and fruit intake a. [file 12877_2022_2939_MOESM3_ESM.docx]

**Table S1.** Amounts of nutrients and foods according to quartiles of vegetable intake at baseline ^a^

| Nutrients and foods | Vegetable intake (g/1,000 kcal) | | | | |
| --- | --- | --- | --- | --- | --- |
|  | Q1 | Q2 | Q3 | Q4 | *P-*trend^c^ |
|  | M: ≤111; W: ≤135  (n=267) | M: 112-151; W: 136-189  (n=268) | M: 152-205; W: 190-250  (n=268) | M: ≥206; W: ≥251  (n=268) |  |
| Vegetable intake (median) |  |  |  |  |  |
| Men (g/1,000 kcal) | 82 | 134 | 177 | 250 |  |
| Women (g/1,000 kcal) | 110 | 163 | 217 | 292 |  |
| ***Nutrient*** |  |  |  |  |  |
| Total dietary fiber, g/1000 kcal | 5.31 (1.53)^b^ | 6.45 (1.87) | 7.20 (1.93) | 8.18 (1.99) | <0.001 |
| Soluble dietary fiber, g/1000 kcal | 0.99 (0.51) | 1.25 (0.72) | 1.33 (0.61) | 1.39 (0.61) | <0.001 |
| Insoluble dietary fiber, g/1000 kcal | 4.32 (1.05) | 5.21 (1.21) | 5.88 (1.37) | 6.79 (1.44) | <0.001 |
| Vitamin A, I.U/1000 kcal | 1,152 (341) | 1605 (355) | 1953 (392) | 2,658 (604) | <0.001 |
| Thiamin, mg/1000 kcal | 0.40 (0.17) | 0.46 (0.22) | 0.49 (0.19) | 0.55 (0.21) | <0.001 |
| Riboflavin, mg/1000 kcal | 0.62 (0.18) | 0.70 (0.18) | 0.71 (0.19) | 0.74 (0.20) | <0.001 |
| Vitamin C, mg/1000 kcal | 31.5 (14.7) | 42.8 (12.2) | 53.8 (12.8) | 73.7 (18.4) | <0.001 |
| Potassium, mg/1000 kcal | 1,058 (227) | 1,280 (207) | 1,420 (235) | 1,694 (303) | <0.001 |
| Calcium, mg/1000 kcal | 288 (105) | 330 (93) | 334 (100) | 359 (101) | <0.001 |
| Magnesium, mg/1000 kcal | 90 (18) | 103 (17) | 110 (21) | 123 (24) | <0.001 |
| ***Food*** |  |  |  |  |  |
| Rice, g/1000 kcal | 124.1 (36.7) | 111.6 (35.1) | 110.7 (35.9) | 113.9 (32.5) | 0.001 |
| Potatoes, g/1000 kcal | 11.8 (15.7) | 11.6 (10.6) | 13.1 (12.2) | 13.9 (15.1) | 0.04 |
| Soybean and soybean products, g/1000 kcal | 44.7 (28.1) | 55.3 (33.7) | 51.3 (32.4) | 52.3 (32.3) | 0.03 |
| Miso, g/1000 kcal | 8.4 (5.0) | 8.7 (4.2) | 8.2 (4.2) | 9.0 (5.0) | 0.33 |
| Green and yellow vegetables, g/1000 kcal | 24 (11) | 40 (14) | 55 (18) | 79 (26) | <0.001 |
| Other vegetables, g/1000 kcal | 54 (20) | 88 (20) | 120 (28) | 175 (50) | <0.001 |
| Pickles, g/1000 kcal | 15.8 (14.8) | 23.1 (20.0) | 25.5 (23.4) | 38.9 (34.2) | <0.001 |
| Fruit, g/1000 kcal | 43 (46) | 47 (40) | 52 (43) | 56 (50) | <0.001 |
| Algae, g/1000 kcal | 0.53 (0.45) | 0.65 (0.50) | 0.71 (0.51) | 0.84 (0.67) | <0.001 |
| Fish, g/1000 kcal | 21.9 (17.4) | 25.4 (17.9) | 22.2 (14.6) | 25.1 (18.6) | 0.19 |
| Meat, g/1000 kcal | 12.4 (10.7) | 12.8 (9.5) | 12.0 (9.2) | 12.1 (12.5) | 0.60 |
| Eggs, g/1000 kcal | 20.5 (14.1) | 21.6 (11.9) | 21.7 (11.9) | 21.0 (13.8) | 0.64 |
| Milk and dairy products, g/1000 kcal | 54.0 (10.7-113.8) | 55.2 (20.4-116.4) | 60.7 (14.3-118.1) | 47.5 (2.8-115.1) | 0.55 |
| Fats and oils, g/1000 kcal | 13.4 (5.1) | 13.0 (4.7) | 12.8 (4.8) | 13.5 (5.0) | 0.86 |
| Sugar and confectioneries, g/1000 kcal | 17.1 (12.1) | 17.0 (12.5) | 16.2 (11.3) | 15.3 (10.3) | 0.05 |
| Alcoholic beverages, g/1000 kcal | 0.0 (0.0-56.7) | 0.0 (0.0-68.2) | 0.0 (0.0-74.0) | 0.0 (0.0-27.0) | 0.003 |
| Salt, g/1000 kcal | 4.62 (2.10) | 4.68 (2.20) | 4.60 (2.26) | 5.28 (2.50) | 0.002 |

^a^The values of milk and dairy products, and alcoholic beverages are shown as the median (interquartile range).

^b^All other values are shown as the mean (standard deviations).

^c^*P*-trend values were calculated from linear regressions using the quartile ordinal as the predictor variable.

**Table S2.** Amounts of nutrients and foods according to quartiles of fruit intake at baseline ^a^

| Nutrients and foods | Fruit intake (g/1,000 kcal) | | | | |
| --- | --- | --- | --- | --- | --- |
|  | Q1 | Q2 | Q3 | Q4 | *P-*trend^c^ |
|  | M: ≤13; W: ≤22  (n=267) | M: 14-30; W: 23-46  (n=268) | M: 31-56; W: 47-71  (n=268) | M: ≥57; W: ≥72  (n=268) |  |
| Fruit intake (median) |  |  |  |  |  |
| Men (g/1,000 kcal) | 4 | 21 | 40 | 88 |  |
| Women (g/1,000 kcal) | 12 | 34 | 54 | 107 |  |
| ***Nutrient*** |  |  |  |  |  |
| Total dietary fiber, g/1000 kcal | 6.17 (2.31)^b^ | 6.64 (2.10) | 6.74 (1.79) | 7.60(1.99) | <0.001 |
| Soluble dietary fiber, g/1000 kcal | 1.08 (0.75) | 1.20 (0.63) | 1.22 (0.53) | 1.45 (0.55) | <0.001 |
| Insoluble dietary fiber, g/1000 kcal | 5.09 (1.66) | 5.44 (1.54) | 5.52 (1.33) | 6.15 (1.52) | <0.001 |
| Vitamin A, I.U/1000 kcal | 1,675 (706) | 1,816 (653) | 1,879 (666) | 2,002 (742) | <0.001 |
| Thiamine, mg/1000 kcal | 0.44 (0.22) | 0.47 (0.20) | 0.50 (0.23) | 0.50 (0.16) | 0.001 |
| Riboflavin, mg/1000 kcal | 0.65 (0.19) | 0.69 (0.21) | 0.70 (0.18) | 0.72 (0.19) | <0.001 |
| Vitamin C, mg/1000 kcal | 39.6 (17.5) | 45.3 (16.8) | 49.7 (17.5) | 66.2 (24.2) | <0.001 |
| Potassium, mg/1000 kcal | 1,225 (315) | 1,332 (330) | 1,377 (288) | 1,519 (345) | <0.001 |
| Calcium, mg/1000 kcal | 307 (108) | 325 (105) | 337 (94) | 341 (102) | <0.001 |
| Magnesium, mg/1000 kcal | 97 (21) | 104 (24) | 108 (21) | 117 (24) | <0.001 |
| ***Food*** |  |  |  |  |  |
| Rice, g/1000 kcal | 121.9 (38.6) | 114.9 (34.7) | 114.5 (33.8) | 108.9 (33.3) | <0.001 |
| Potatoes, g/1000 kcal | 10.3 (16.2) | 13.1 (12.1) | 12.8 (11.2) | 14.1 (13.5) | 0.002 |
| Soybean and soybean products, g/1000 kcal | 50.5 (35.0) | 52.6 (32.3) | 50.0 (30.0) | 50.5 (30.2) | 0.78 |
| Miso, g/1000 kcal | 8.9 (4.8) | 9.0 (5.0) | 8.3 (4.0) | 8.1 (4.5) | 0.01 |
| Vegetable, g/1000 kcal | 177 (80) | 210 (80) | 235 (81) | 313 (101) | <0.001 |
| Green and yellow vegetables, g/1000 kcal | 44 (25) | 48 (27) | 50 (25) | 55 (30) | <0.001 |
| Other vegetables, g/1000 kcal | 100 (52) | 107 (53) | 108 (53) | 122 (58) | <0.001 |
| Pickles, g/1000 kcal | 24.9 (27.1) | 25.2 (24.5) | 27.4 (25.7) | 25.7 (25.0) | 0.52 |
| Algae, g/1000 kcal | 0.57 (0.48) | 0.73 (0.55) | 0.70 (0.52) | 0.74 (0.62) | 0.002 |
| Fish, g/1000 kcal | 24.2 (21.5) | 23.5 (15.2) | 24.2 (16.1) | 22.7 (15.6) | 0.45 |
| Meat, g/1000 kcal | 12.1 (11.0) | 12.6 (9.5) | 11.7 (9.2) | 13.0 (12.2) | 0.53 |
| Eggs, g/1000 kcal | 20.8 (14.5) | 22.1 (13.0) | 20.8 (12.4) | 21.1 (12.0) | 0.88 |
| Milk and dairy products, g/1000 kcal | 36.2 (0.9-91.3) | 54.8 (11.9-114.5) | 72.1 (21.3-125.4) | 69.5 (15.9-116.4) | 0.01 |
| Fats and oils, g/1000 kcal | 12.9 (5.3) | 13.6 (5.0) | 13.0 (4.8) | 13.2 (4.4) | 0.94 |
| Sugar and confectioneries, g/1000 kcal | 15.4 (11.8) | 16.2 (11.6) | 16.6 (11.5) | 17.4 (11.5) | 0.05 |
| Alcoholic beverages, g/1000 kcal | 0.0 (0.0-87.8) | 0.0 (0.0-55.5) | 0.0 (0.0-55.0) | 0.0 (0.0-22.9) | 0.05 |
| Salt, g/1000 kcal | 5.14 (2.60) | 4.80 (2.33) | 4.66 (2.14) | 4.59 (2.01) | 0.004 |

^a^ The values of milk and dairy products, and alcoholic beverages are shown as the median (interquartile range).

^b^ All other values are shown as the mean (standard deviations).

^c^ *P*-trend values were calculated from linear regressions using the quartile ordinal as the predictor variable.

**Table S3.** Spearman’s correlation coefficients between nutrients and intake of vegetables and fruits

| Nutrient | Vegetable | Fruit |
| --- | --- | --- |
| Total energy | -0.16 | -0.03 |
| Protein | 0.19 | 0.08 |
| Fat | 0.04 | 0.04 |
| Carbohydrate | 0.05 | 0.14 |
| Total dietary fiber | 0.58^b^ | 0.31^a^ |
| Soluble dietary fiber | 0.29 | 0.29 |
| Insoluble dietary fiber | 0.67^b^ | 0.31^a^ |
| Vitamin A | 0.87^b^ | 0.20 |
| Thiamin | 0.63^b^ | 0.32^a^ |
| Riboflavin | 0.25 | 0.16 |
| Vitamin C | 0.86^b^ | 0.49^a^ |
| Potassium | 0.76^b^ | 0.36^a^ |
| Calcium, | 0.27 | 0.15 |
| Magnesium | 0.59^b^ | 0.36^a^ |

^a^ Weak positive correlation (correlation coefficient of 0.30 to 0.49).

^b^ Moderate or strong positive correlation (correlation coefficient of ≥0.50).

| **Table S4.** Multivariable-adjusted hazard ratios of total dementia per 1SD increment in vegetable intake between subgroups of age, sex, education levels and current smoking status in the study population, 1988-2012 | | | | |
| --- | --- | --- | --- | --- |
| Variables | Number of events/subjects | Hazard ratio  (95% confidence interval) ^a^ | *P* value | *P* for interaction |
| Age |  |  |  |  |
| <75 years | 355/807 | 0.92 (0.82-1.03) | 0.13 | 0.59 |
| ≥75 years | 109/253 | 0.84 (0.67-1.07) | 0.16 |  |
| Sex |  |  |  |  |
| Men | 151/446 | 0.95 (0.79-1.14) | 0.57 | 0.31 |
| Women | 313/614 | 0.89 (0.79-1.01) | 0.07 |  |
| Education |  |  |  |  |
| ≤6 years | 70/133 | 0.73 (0.54-0.98) | 0.03 | 0.55 |
| >6years | 394/927 | 0.91 (0.81-1.02) | 0.09 |  |
| Curremt Smoking |  |  |  |  |
| No | 372/816 | 0.88 (0.79-0.99) | 0.03 | 0.45 |
| Yes | 92/244 | 0.97 (0.77-1.23) | 0.80 |  |
| ^a^ Adjusted for age, educational level, history of stroke, diabetes, systolic blood pressure, use of antihypertensive agent, electrocardiogram abnormalities, total cholesterol, body mass index, current drinking, current smoking, regular exercise, intakes of total energy, protein, fat, and carbohydrate. | | | | |

**Table S5.** Adjusted hazard ratios of total dementia according to quartiles of the total consumption of the vegetable and fruit intake^a^

| Food intake levels  (g/day) | Number of  events/ PYs | Age- and sex-adjusted incidence  (per 10^3^ PYs) | Hazard ratio (95% CI) | |
| --- | --- | --- | --- | --- |
|  |  |  | Age- and sex-adjusted | Multivariable-adjusted^b, c^ |
| **Vegetable + Fruit** |  |  |  |  |
| Q1 (M: ≤146; W: ≤183) | 115/3,580 | 33.6 | 1.00 (reference) | 1.00 (reference) |
| Q2 (M: 147-194; W: 184-241) | 127/3,711 | 34.6 | 1.16 (0.90, 1.50) | 1.17 (0.90, 1.52) |
| Q3 (M: 195-259; W: 242-311) | 107/3,869 | 28.3 | 0.79 (0.60, 1.03) | 0.79 (0.60, 1.04) |
| Q4 (M: ≥260; W: ≥312) | 115/3,991 | 29.2 | 0.81 (0.62, 1.05) | 0.85 (0.65, 1.13) |
| *P-*trend |  |  | 0.02 | 0.046 |

^a^ M, men; W, women; PYs, person-years; Q, quartile.

^b^ Adjusted for age, sex, educational level, history of stroke, diabetes, systolic blood pressure, use of antihypertensive agents, electrocardiogram abnormalities, total cholesterol, body mass index, current drinking, current smoking, regular exercise, and intakes of total energy, protein, fat, and carbohydrate.

^c^ n = 1,060

**Table S6.** Adjusted hazard ratios of Alzheimer's disease and vascular dementia according to quartiles of the total consumption of the vegetable and fruit intake ^a^

| Food intake levels  (g/1,000 kcal) | Alzheimer’s disease | | | |  | Vascular dementia | | | |
| --- | --- | --- | --- | --- | --- | --- | --- | --- | --- |
|  | Number of events/ PYs | Age- and sex-adjusted incidence  (per 10^3^ PYs) | Hazard ratio (95% CI) | |  | Number of events/ PYs | Age- and sex-adjusted incidence  (per 10^3^ PYs) | Hazard ratio (95% CI) | |
|  |  |  | Age- and sex-adjusted | Multivariable-adjusted ^b, c^ |  |  |  | Age- and sex-adjusted | Multivariable-adjusted^b, c^ |
| **Vegetable + Fruit** |  |  |  |  |  |  |  |  |  |
| Q1 (M: ≤146; W: ≤183) | 77/3,580 | 21.3 | 1.00 (reference) | 1.00 (reference) |  | 31/3,580 | 9.2 | 1.00 (reference) | 1.00 (reference) |
| Q2 (M: 147-194; W: 184-241) | 66/3,711 | 16.9 | 0.90 (0.65, 1.26) | 0.91 (0.65, 1.28) |  | 53/3,711 | 14.8 | 1.77 (1.13, 2.76) | 1.94 (1.23, 3.07) |
| Q3 (M: 195-259; W: 242-311) | 64/3,869 | 16.0 | 0.71 (0.51, 0.99) | 0.70 (0.50, 0.99) |  | 33/3,869 | 8.8 | 0.92 (0.57, 1.51) | 0.99 (0.60, 1.64) |
| Q4 (M: ≥260; W: ≥312) | 79/3,991 | 18.9 | 0.83 (0.60, 1.13) | 0.90 (0.64, 1.26) |  | 27/3,991 | 6.9 | 0.75 (0.45, 1.25) | 0.73 (0.42, 1.26) |
| *P-*trend |  |  | 0.13 | 0.27 |  |  |  | 0.04 | 0.047 |

^a^ M, men; W, women; PYs, person-years; Q, quartile.

^b^ Adjusted for age, sex, educational level, history of stroke, diabetes, systolic blood pressure, use of antihypertensive agent, electrocardiogram abnormalities, total cholesterol, body mass index, current drinking, current smoking, regular exercise, and intakes of total energy, protein, fat, and carbohydrate.

^c^ n = 1,060

**Table S7.** Adjusted hazard ratios of total dementia according to quartiles of vegetable and fruit intake with the exclusion of subjects developing dementia within a follow-up period of 2 years or less^a^

| Food intake levels  (g/1,000 kcal) | Number of  events/ PYs | Age- and sex-adjusted incidence  (per 10^3^ PYs) | Hazard ratio (95% CI) | |
| --- | --- | --- | --- | --- |
|  |  |  | Age- and sex-adjusted | Multivariable-adjusted^b, c^ |
| **Vegetable** |  |  |  |  |
| Q1 (M: ≤112; W: ≤135) | 115/3,573 | 33.5 | 1.00 (reference) | 1.00 (reference) |
| Q2 (M: 113-152; W: 136-189) | 114/3,857 | 30.1 | 0.82 (0.63, 1.06) | 0.85 (0.65, 1.11) |
| Q3 (M: 153-205; W: 190-250) | 107/3,728 | 28.8 | 0.81 (0.62, 1.05) | 0.87 (0.66, 1.14) |
| Q4 (M: ≥206; W: ≥251) | 111/3,976 | 28.2 | 0.72 (0.56, 0.94) | 0.73 (0.55, 0.96) |
| *P-*trend |  |  | 0.02 | 0.04 |
| **Fruit** |  |  |  |  |
| Q1 (M: ≤13; W: ≤22) | 113/3,582 | 32.7 | 1.00 (reference) | 1.00 (reference) |
| Q2 (M: 14-31; W: 23-46) | 112/3,825 | 29.9 | 0.84 (0.64, 1.09) | 0.87 (0.67, 1.14) |
| Q3 (M: 32-56; W: 47-71) | 104/3,867 | 27.2 | 0.74 (0.57, 0.97) | 0.77 (0.58, 1.01) |
| Q4 (M: ≥57; W: ≥72) | 118/3,859 | 30.8 | 0.87 (0.67, 1.12) | 0.92 (0.70, 1.20) |
| *P-*trend |  |  | 0.22 | 0.39 |

^a^ M, men; W, women; PYs, person-years; Q, quartile.

^b^ Adjusted for age, sex, educational level, history of stroke, diabetes, systolic blood pressure, use of antihypertensive agents, electrocardiogram abnormalities, total cholesterol, body mass index, current drinking, current smoking, regular exercise, and intakes of total energy, protein, fat, and carbohydrate.

^c^ n = 1,060

**Table S8.** Adjusted hazard ratios of Alzheimer's disease and vascular dementia according to quartiles of vegetable and fruit intake with the exclusion of subjects developing dementia within a follow-up period of 2 years or less^a^

| Food intake levels  (g/1,000 kcal) | Alzheimer’s disease | | | |  | Vascular dementia | | | |
| --- | --- | --- | --- | --- | --- | --- | --- | --- | --- |
|  | Number of events/ PYs | Age- and sex-adjusted incidence  (per 10^3^ PYs) | Hazard ratio (95% CI) | |  | Number of events/ PYs | Age- and sex-adjusted incidence  (per 10^3^ PYs) | Hazard ratio (95% CI) | |
|  |  |  | Age- and sex-adjusted | Multivariable-adjusted ^b, c^ |  |  |  | Age- and sex-adjusted | Multivariable-adjusted ^b, c^ |
| **Vegetable** |  |  |  |  |  |  |  |  |  |
| Q1 (M: ≤112; W: ≤135) | 74/3,573 | 20.5 | 1.00 (reference) | 1.00 (reference) |  | 33/3,573 | 9.7 | 1.00 (reference) | 1.00 (reference) |
| Q2 (M: 113-152; W: 136-189) | 70/3,857 | 17.3 | 0.75 (0.54, 1.04) | 0.73 (0.52, 1.02) |  | 41/3,857 | 11.1 | 1.09 (0.69, 1.73) | 1.26 (0.79, 2.02) |
| Q3 (M: 153-205; W: 190-250) | 63/3,728 | 16.0 | 0.73 (0.52, 1.02) | 0.73 (0.52, 1.03) |  | 36/3,728 | 9.8 | 0.97 (0.61, 1.56) | 1.21 (0.74, 1.97) |
| Q4 (M: ≥206; W: ≥251) | 72/3,978 | 17.2 | 0.69 (0.50, 0.96) | 0.67 (0.47, 0.95) |  | 31/3,976 | 8.0 | 0.76 (0.47, 1.25) | 0.77 (0.46, 1.29) |
| *P-*trend |  |  | 0.04 | 0.04 |  |  |  | 0.22 | 0.30 |
| **Fruit** |  |  |  |  |  |  |  |  |  |
| Q1 (M: ≤13; W: ≤22) | 67/3,582 | 18.3 | 1.00 (reference) | 1.00 (reference) |  | 44/3,582 | 12.9 | 1.00 (reference) | 1.00 (reference) |
| Q2 (M: 14-31; W: 23-46) | 58/3,825 | 14.4 | 0.71 (0.50, 1.01) | 0.73 (0.51, 1.05) |  | 37/3,825 | 10.2 | 0.74 (0.48, 1.15) | 0.77 (0.49, 1.20) |
| Q3 (M: 32-56; W: 47-71) | 73/3,867 | 18.1 | 0.89 (0.64, 1.24) | 0.88 (0.63, 1.23) |  | 29/3,867 | 7.6 | 0.54 (0.34, 0.86) | 0.63 (0.39, 1.02) |
| Q4 (M: ≥57; W: ≥72) | 81/3,859 | 20.0 | 1.02 (0.74, 1.42) | 1.06 (0.76, 1.48) |  | 31/3,859 | 8.2 | 0.58 (0.37, 0.92) | 0.65 (0.40, 1.04) |
| *P-*trend |  |  | 0.52 | 0.48 |  |  |  | 0.01 | 0.051 |

^a^ M, men; W, women; PYs, person-years; Q, quartile.

^b^ Adjusted for age, sex, educational level, history of stroke, diabetes, systolic blood pressure, use of antihypertensive agents, electrocardiogram abnormalities, total cholesterol, body mass index, current drinking, current smoking, regular exercise, and intakes of total energy, protein, fat, and carbohydrate.

^c^ n = 1,060

**Table S9.** Baseline characteristics of potential risk factors for dementia according to quartiles of the absolute values of the vegetable and fruit intake ^a^

| Characteristic | Vegetable intake (g/day) | | | | | Fruit intake (g/day) | | | |
| --- | --- | --- | --- | --- | --- | --- | --- | --- | --- |
|  | Q1 | Q2 | Q3 | Q4 |  | Q1 | Q2 | Q3 | Q4 |
|  | M: ≤194  W: ≤198  (n=267) | M: 195-277  W: 199-281  (n=268) | M: 278-362  W: 282-361  (n=269) | M: ≥363  W: ≥362  (n=267) |  | M: ≤21  W: ≤32  (n=300) | M: 22-56  W: 33-74  (n=205) | M: 57-99  W: 75-114  (n=298) | M: ≥100  W: ≥115  (n=268) |
| Vegetable or fruit intake (median) |  |  |  |  |  |  |  |  |  |
| Men, g/day | 150 | 240 | 316 | 450 |  | 11 | 43 | 75 | 150 |
| Women, g/day | 154 | 240 | 314 | 450 |  | 21 | 43 | 75 | 150 |
| ***Clinical parameters*** |  |  |  |  |  |  |  |  |  |
| Age, y | 70.1 (6.7)^b^ | 69.5 (6.6) | 69.3 (6.5) | 68.9 (6.2) |  | 69.7 (6.8) | 69.6 (6.9) | 69.2 (6.3) | 69.3 (6.1) |
| Men, % | 42.3 | 42.2 | 42.4 | 42.0 |  | 41.7 | 49.8 | 37.6 | 42.2 |
| Education ≤6 years, %^c^ | 17.4 | 14.1 | 12.0 | 6.8 |  | 16.1 | 12.5 | 14.6 | 6.3 |
| History of stroke, % | 4.5 | 5.6 | 3.7 | 3.8 |  | 5.0 | 6.3 | 3.4 | 3.4 |
| Diabetes mellitus, % | 13.1 | 13.8 | 17.1 | 15.0 |  | 14.3 | 16.6 | 13.1 | 15.7 |
| Body mass index, kg/m^2^ | 22.0 (3.1) | 22.3 (3.1) | 22.1 (3.1) | 22.9 (2.9) |  | 21.9 (3.1) | 22.2 (2.9) | 22.4 (3.2) | 22.7 (3.1) |
| Systolic blood pressure, mmHg | 139 (22) | 138 (23) | 139 (24) | 139 (20) |  | 139 (21) | 139 (23) | 138 (23) | 140 (22) |
| Diastolic blood pressure, mmHg | 76 (11) | 76 (10) | 76 (11) | 76 (11) |  | 76 (11) | 76 (11) | 75 (10) | 77 (10) |
| Antihypertensive agents, % | 23.6 | 28.7 | 24.2 | 22.5 |  | 24.0 | 24.9 | 24.2 | 26.1 |
| Electrocardiogram abnormalities, % | 20.6 | 20.2 | 17.8 | 22.5 |  | 23.0 | 21.0 | 17.1 | 20.2 |
| Serum total cholesterol, mg/dL | 208 (45) | 210 (45) | 206 (42) | 212 (45) |  | 204 (44) | 204 (46) | 214 (44) | 212 (43) |
| Current smoking, % | 26.6 | 22.4 | 23.8 | 19.9 |  | 30.0 | 29.3 | 17.8 | 16.8 |
| Current alcohol drinking, % | 24.3 | 25.8 | 29.4 | 25.1 |  | 33.0 | 25.9 | 24.2 | 20.9 |
| Regular exercise, % | 12.0 | 15.3 | 13.8 | 18.4 |  | 13.3 | 15.1 | 17.8 | 13.1 |
| ***Dietary factors*** |  |  |  |  |  |  |  |  |  |
| Total energy, kcal/day | 1,465 (353) | 1,621 (362) | 1,621 (357) | 1,782 (376) |  | 1,540 (397) | 1,577 (380) | 1,658 (366) | 1,708 (347) |
| Protein, g/day | 45.8 (14.6) | 53.5 (15.0) | 54.4 (16.1) | 62.9 (17.6) |  | 50.5 (17.6) | 51.4 (16.7) | 56.4 (16.4) | 57.7 (15.9) |
| Fat, g/day | 41.5 (13.6) | 46.9 (13.2) | 46.5 (14.6) | 51.8 (16.3) |  | 43.5 (15.5) | 45.2 (13.9) | 48.7 (15.1) | 49.1 (13.9) |
| Carbohydrate, g/day | 206.4 (51.1) | 224.1 (54.5) | 222.2 (49.3) | 243.6 (53.5) |  | 211.4 (56.0) | 217.4 (50.2) | 226.1 (51.6) | 241.3 (51.4) |

^a^ M, men; W, women; Q, quartile.

^b^ Values are expressed as the mean (standard deviation) or frequency.

^c^ n = 1,060

**Table S10.** Adjusted hazard ratios of total dementia according to quartiles of the absolute values of the vegetable and fruit intake^a^

| Food intake levels  (g/day) | Number of  events/ PYs | Hazard ratio (95% CI) | |
| --- | --- | --- | --- |
|  |  | Age- and sex-adjusted | Multivariable-adjusted^b, c^ |
| **Vegetable** |  |  |  |
| Q1 (M: ≤194; W: ≤198) | 113/3,386 | 1.00 (reference) | 1.00 (reference) |
| Q2 (M: 195-277; W: 199-281) | 120/3,654 | 0.89 (0.68, 1.15) | 1.00 (0.76, 1.30) |
| Q3 (M: 278-362; W: 282-361) | 118/3,987 | 0.83 (0.60, 1.02) | 0.82 (0.63, 1.08) |
| Q4 (M: ≥363; W: ≥362) | 113/4,129 | 0.66 (0.51, 0.87) | 0.76 (0.57, 1.02) |
| *P-*trend |  | 0.01 | 0.03 |
| **Fruit** |  |  |  |
| Q1 (M: ≤21; W: ≤32) | 133/3,951 | 1.00 (reference) | 1.00 (reference) |
| Q2 (M: 22-56; W: 33-74) | 83/2,768 | 0.92 (0.69, 1.11) | 0.99 (0.74, 1.32) |
| Q3 (M: 57-99; W: 75-114) | 129/4,456 | 0.72 (0.56, 0.92) | 0.81 (0.63, 1.04) |
| Q4 (M: ≥100; W: ≥115) | 119/3,976 | 0.77 (0.60, 0.99) | 0.93 (0.71, 1.22) |
| *P-*trend |  | 0.01 | 0.32 |

^a^ M, men; W, women; PYs, person-years; Q, quartile.

^b^ Adjusted for age, sex, educational level, history of stroke, diabetes, systolic blood pressure, use of antihypertensive agents, electrocardiogram abnormalities, total cholesterol, body mass index, current drinking, current smoking, regular exercise, and intakes of total energy, protein, fat, and carbohydrate.

^c^ n = 1,060

**Table S11.** Adjusted hazard ratios of Alzheimer's disease and vascular dementia according to quartiles of the absolute values of the vegetable and fruit intake ^a^

| Food intake levels  (g/day) | Alzheimer’s disease | | | |  | Vascular dementia | | | |
| --- | --- | --- | --- | --- | --- | --- | --- | --- | --- |
|  | Number of events/ PYs | Age- and sex-adjusted incidence  (per 10^3^ PYs) | Hazard ratio (95% CI) | |  | Number of events/ PYs | Age- and sex-adjusted incidence  (per 10^3^ PYs) | Hazard ratio (95% CI) | |
|  |  |  | Age- and sex-adjusted | Multivariable-adjusted^b, c^ |  |  |  | Age- and sex-adjusted | Multivariable-adjusted^b, c^ |
| **Vegetable** |  |  |  |  |  |  |  |  |  |
| Q1 (M: ≤194; W: ≤198) | 75/3,385 | 21.1 | 1.00 (reference) | 1.00 (reference) |  | 32/3,385 | 9.5 | 1.00 (reference) | 1.00 (reference) |
| Q2 (M: 195-277; W: 199-281) | 67/3,652 | 17.5 | 0.70 (0.50, 0.98) | 0.79 (0.56, 1.11) |  | 46/3,652 | 12.9 | 1.27 (0.81, 2.00) | 1.38 (0.86, 2.21) |
| Q3 (M: 278-362; W: 282-361) | 74/3,986 | 17.8 | 0.66 (0.47, 0.91) | 0.70 (0.50, 0.98) |  | 37/3,986 | 9.8 | 0.94 (0.59, 1.51) | 1.09 (0.67, 1.79) |
| Q4 (M: ≥363; W: ≥362) | 70/4,128 | 16.7 | 0.54 (0.39, 0.76) | 0.66 (0.45, 0.95) |  | 29/4,128 | 7.5 | 0.71 (0.43, 1.18) | 0.77 (0.44, 1.33) |
| *P-*trend |  |  | 0.01 | 0.02 |  |  |  | 0.08 | 0.21 |
| **Fruit** |  |  |  |  |  |  |  |  |  |
| Q1 (M: ≤21; W: ≤32) | 77/3,951 | 18.8 | 1.00 (reference) | 1.00 (reference) |  | 48/3,951 | 12.5 | 1.00 (reference) | 1.00 (reference) |
| Q2 (M: 22-56; W: 33-74) | 45/2,768 | 16.2 | 0.83 (0.57, 1.20) | 0.90 (0.62, 1.32) |  | 28/2,768 | 10.6 | 0.90 (0.56, 1.43) | 1.00 (0.62, 1.62) |
| Q3 (M: 57-99; W: 75-114) | 86/4,456 | 18.2 | 0.78 (0.57, 1.06) | 0.86 (0.63, 1.19) |  | 33/4,456 | 7.8 | 0.56 (0.36, 0.88) | 0.72 (0.45, 1.15) |
| Q4 (M: ≥100; W: ≥115) | 78/3,976 | 19.0 | 0.88 (0.64, 1.22) | 1.09 (0.77, 1.54) |  | 35/3,976 | 9.1 | 0.67 (0.44, 1.04) | 0.86 (0.54 , 1.37) |
| *P-*trend |  |  | 0.38 | 0.80 |  |  |  | 0.02 | 0.31 |

^a^ M, men; W, women; PYs, person-years; Q, quartile.

^b^ Adjusted for age, sex, educational level, history of stroke, diabetes, systolic blood pressure, use of antihypertensive agent, electrocardiogram abnormalities, total cholesterol, body mass index, current drinking, current smoking, regular exercise, and intakes of total energy, protein, fat, and carbohydrate.

^c^ n = 1,060
